# Alcohol Consumption and Mortality in Japan: The Miyagi Cohort Study

**DOI:** 10.2188/jea.14.S18

**Published:** 2005-03-18

**Authors:** Naoki Nakaya, Kayoko Kurashima, Junko Yamaguchi, Takayoshi Ohkubo, Yoshikazu Nishino, Yoshitaka Tsubono, Daisuke Shibuya, Shin Fukudo, Akira Fukao, Ichiro Tsuji, Shigeru Hisamichi

**Affiliations:** 1 Division of Epidemiology, Department of Public Health and Forensic Medicine, Tohoku University Graduate School of Medicine.; 2 Miyagi Cancer Society.; 3 Department of Behavioral Medicine, Tohoku University Graduate School of Medicine.; 4 Department of Public Health, Yamagata University School of Medicine.

**Keywords:** alcohol consumption, all-cause mortality, prospective cohort study

## Abstract

BACKGROUND: We examined the association between alcohol consumption and all-cause mortality in Japanese men and women.

METHODS: From June through August 1990, a total of 39,076 subjects (20,660 men and 18,416 women) in 14 municipalities of Miyagi Prefecture in rural northern Japan (40-64 years of age) completed a self-administered questionnaire that included information about alcohol consumption and various health habits. During 11 years of follow-up, we identified 1,879 deaths (1,335 men and 544 women). We used Cox proportional-hazards regression to estimate relative risk (RR) of all-cause mortality according to categories of alcohol consumption and to adjust for age, education, marital status, past histories of chronic diseases, body mass index, smoking, walking and dietary variables.

RESULTS: Among men, the risk for all-cause mortality was significantly higher in past drinkers than never-drinkers (multivariate RR, 1.86; 95% confidence interval [CI], 1.50-2.29). There was a dose-response association between alcohol consumption and the risk of all-cause mortality among current drinking men: multivariate RRs in reference to never-drinkers (95% CI) were 1.10 (0.90-1.33), 1.17 (0.96-1.42), 1.16 (0.96-1.40), and 1.62 (1.32-1.99) in current drinkers who consumed less than 22.8 g, 22.8-45.5 g, 45.6-68.3 g, and 68.4 g or more alcohol per day, respectively (P for trend<0.001). Similar association was observed among women (P for trend=0.005).

CONCLUSIONS: The results indicated that alcohol consumption tended to be associated with linear increase in risk of all-cause mortality among Japanese men and women, and the association was remarkable for younger men.

Epidemiologic studies of alcohol consumption and all-cause mortality among men have been inconsistent. Although many studies showed a J-shaped^1^^-^^11^ or U-shaped^12^^-^^18^ association, indicating a protective effect of moderate alcohol consumption, some studies found linear,^19^^-^^22^ no,^23^^-^^25^ or other^26^^-^^32^ associations. The results were inconsistent among Japanese studies: four prospective cohort studies found J-shaped,^11^ linear,^22^ or other associations.^27^^,^^32^

Fewer studies were available for women than for men, and the findings have also been inconsistent. Some studies reported a J-shaped^6^^,^^10^^,^^18^^,^^33^ or U-shaped^34^ associations, while others reported no^28^^,^^31^ or other^16^ associations between alcohol consumption and all-cause mortality. To our knowledge, no studies have been reported for the Japanese women.

We have previously conducted a prospective cohort study among middle-aged men in rural northern Japan who were followed-up for 7 years, and reported that current drinkers had linear increase in risk of all-cause mortality associated with higher amount of consumption as compared with never-drinkers.^22^ Here we report the findings from the same cohort with 11 years of follow-up for men and women.

## METHODS

### Study Cohort

We have reported the design of this prospective cohort study in detail elsewhere.^35^ Briefly, from June through August 1990, we delivered a self-administered questionnaire on various health habits to 51,921 subjects (25,279 men and 26,642 women) who were 40-64 years of age and lived in 14 municipalities of Miyagi Prefecture in northern Japan. The questionnaires were delivered to and collected from the subjects’ residences by members of health promotion committees appointed by the municipal governments. Usable questionnaires were returned from 47,605 subjects (22,836 men and 24,769 women), yielding a response rate of 91.7%.

The study protocol was approved by the institutional review board of Tohoku University Graduate School of Medicine. We considered the return of the self-administered questionnaires signed by the subjects to imply their consent to participate in the study.

### Exposure Data

For the assessment of alcohol consumption, the questionnaire asked firstly if subjects were current, past, or never drinkers. Current drinkers were further asked about drinking frequency (less than once per week, once or twice per week, three or four times per week, or five times or more per week), beverage type usually consumed (sake, spirits, beer, whisky, wine, or others), and amount at one occasion. From these data, we calculated the grams of alcohol consumed per day, and classified the men who were current drinkers into four categories (< 22.8 g, 22.8-45.5 g, 45.6-68.3 g, or 68.4 g or more alcohol per day) and the women who were current drinkers into two categories (< 22.8 g or 22.8 g or more alcohol per day). With regard to the alcohol contents, 22.8 g of alcohol amounts to 1 go or traditional unit of sake (180 ml), which approximates two glasses of wine (200 ml), or two measures of spirits (50 ml).

We conducted a validation study for the questionnaire assessment of alcohol consumption.^36^ Specifically, 113 subjects in the study district completed the questionnaire twice with a 12-month interval and provided four three-day diet records during the 12 months. Spearman’s coefficients for the correlation between the amounts of alcohol consumed according to the questionnaire and the amounts consumed according to the diet records were 0.70 for men and 0.60 for women, and the correlations between consumption measured by the two questionnaires administered 12 months apart were 0.76 for men and 0.66 for women.

### Follow-up

Of 47,605 subjects who responded to the questionnaire, we excluded 1,522 subjects who indicated that they had prior histories of cancer (n=561), stroke (n=379), or myocardial infarction (n=582). We also excluded 539 subjects who had prevalent cancer, which we ascertained by record linkage to the population-based cancer registry covering the study area.^37^ We further excluded subjects who had incomplete responses for alcohol information (n=6,468). Consequently, 39,076 subjects (20,660 men and 18,416 women) with 1,879 deaths (1,335 men and 544 women) were included in this analysis.

We followed up vital and residential status of subjects from June 1, 1990, through March 31, 2001. For this follow-up, we established the Follow-up Committee that was consisted of Miyagi Cancer Society; Community Health Division of all 14 municipalities; Department of Health and Welfare, Miyagi Prefectural Government; and Division of Epidemiology, Tohoku University Graduate School of Medicine. The Committee periodically reviewed the Residential Registration Record of each municipality. With this review, we identified the subjects who either died or emigrated during observation. For both decedents and emigrants, we recorded the date of death or emigration. For decedents, we investigated cause of death by reviewing the death certificates of the subjects at Public Health Centers of the study area. The underlying cause of death was coded according to International Classification of Diseases, the Ninth Revision (ICD9). We discontinued follow-up of subjects who emigrated from the study municipalities because of logistical limitations.

We counted person-years of follow-up for each subject from June 1, 1990, until the date of death, date of emigration outside the study districts, or the end of the study period (March 31, 2001), whichever occurred first. A total of 404,168 person-years accrued. There were 1,852 subjects (4.7% of the analytic cohort) who emigrated from the study municipalities and were lost to follow-up.

### Statistical Analysis

We used Cox proportional-hazards regression to estimate relative risk (RR) of all-cause mortality according to categories of alcohol consumption and to adjust for potentially confounding variables, using the PHREG procedure on SAS^®^ version 8.2 statistical software package (SAS Inc., Cary, NC, USA). We conducted all analyses separately for men and women.

We considered the following variables as potential confounders: age in years; education (up to 15 years of age, 16-18, or 19 years or older); marital status at baseline (whether or not living with spouse); past histories of hypertension, renal diseases, liver diseases, diabetes mellitus, peptic ulcers, or tuberculosis; cigarette smoking (never smokers, past smokers, current smokers smoking 1-19 cigarettes per day, or current smokers smoking at least 20 cigarettes per day); body mass index in kg/m^2^ (less than 18.5, 18.5-24.9, or 25.0 or higher); walking time per day (less than 1 hour, or 1 hour or longer); and consumption frequencies of green vegetables and oranges (almost daily, 3-4 times per week, 1-2 times per week, or 1-2 times per month or less often).

We repeated all analyses after excluding the subjects who died during the first three years of follow-up. We also conducted stratified analyses according to the categories of covariates included in the multivariate analyses to examine whether the association between alcohol consumption and all-cause mortality was modified by these variables. P values for tests of linear trends were estimated using grams of alcohol consumed per day as a continuous variable, with the exclusion of past drinkers. All P values were two-tailed.

## RESULTS

The proportions of never drinkers, past drinkers, and current drinkers among men were 15.9%, 7.0%, and 77.1%, respectively, and these proportions among women were 72.1%, 4.0%, and 23.9%, respectively. Table 1 compares the characteristics of subjects according to categories of alcohol consumption. Compared with men who never drank alcohol, men who currently drank alcohol were younger, more likely to be current smokers smoking at least 20 cigarettes per day, less likely to consume oranges daily, and more likely to have histories of hypertension and liver diseases. Compared with women who never drank alcohol, women who currently drank alcohol were younger and more likely to be current smokers.

**Table 1.** Characteristics of subjects according to alcohol categories.

	Men							Women				
	
	Never drinkers	Past drinkers	Current drinkers (g alcohol / day)					Never drinkers	Past drinkers	Current drinkers (g alcohol / day)		
	
			All	<22.8	22.8 - 45.5	45.6 - 68.3	≥68.4			All	<22.8	≥22.8
No. of subjects	3,276	1,448	15,936	4,821	3,820	4,576	2,719	13,283	740	4,393	3,837	556
Mean age (SD)	52.3 (7.7)	54.5 (7.3)	51.0 (7.5)	50.6 (7.6)	51.5 (7.7)	51.6 (7.5)	50.2 (7.0)	52.4 (7.4)	51.5 (7.4)	49.1 (7.1)	49.0 (7.1)	49.5 (6.9)
Body Mass Index (%)												
≤18.5	2.6	3.3	1.8	1.6	1.7	1.8	2.1	2.8	3.9	2.6	2.6	2.6
18.5-24.9	71.0	68.0	70.6	69.6	71.0	71.9	69.9	66.1	64.2	68.9	68.9	68.5
25.0≤	26.4	28.8	27.6	28.8	27.3	26.4	28.0	31.1	31.9	28.5	28.5	28.8
Education (%)												
≤15	44.0	50.0	39.1	37.3	37.0	40.8	40.2	39.4	43.3	33.8	33.0	39.0
16-18	42.9	38.6	46.3	45.9	48.4	45.5	46.3	47.4	45.3	50.7	51.0	48.3
19≤	13.1	11.4	14.6	16.8	14.6	13.7	13.6	13.2	11.4	15.5	15.9	12.6
Living with spouse (%)												
Yes	90.6	91.0	93.1	92.5	93.4	93.9	92.7	87.6	83.0	86.7	87.1	83.4
No	9.4	9.0	6.9	7.5	6.6	6.1	7.3	12.4	17.0	13.3	12.9	16.6
Past history (%)												
Hypertension	10.8	23.6	18.7	13.9	19.4	20.5	23.0	20.0	22.4	16.6	16.0	21.2
Renal diseases	2.9	5.6	2.9	3.2	2.8	2.6	3.3	3.8	5.7	4.5	4.5	4.1
Liver diseases	4.5	15.2	6.0	4.9	5.7	5.8	8.9	3.5	6.8	3.3	3.1	4.7
Diabetes mellitus	4.5	9.8	4.9	4.5	4.4	4.4	7.1	3.1	5.3	1.9	2.0	1.3
Peptic ulcers	20.3	26.5	19.9	20.3	20.6	19.5	18.8	9.1	14.7	10.4	10.3	11.3
Tuberculosis	3.4	4.4	2.9	2.7	3.2	3.0	2.6	2.3	3.2	3.0	2.9	3.4
Green vegetables (%)												
<1-2 tinies/montli	16.0	16.5	14.8	14.2	13.2	15.0	17.9	7.9	13.8	9.1	8.6	12.6
1-2 times/week	35.8	31.4	35.3	35.0	34.3	35.7	36.7	28.3	30.2	32.8	33.0	31.7
3-4 times/week	28.4	30.6	29.8	30.4	31.5	29.1	27.6	36.3	32.4	35.1	35.3	33.5
Everyday	19.8	21.5	20.0	20.4	20.9	20.2	17.8	27.5	23.6	23.0	23.1	22.2
Oranges (%)												
<1-2 tinies/montli	25.3	27.9	30.8	25.4	28.9	34.1	37.6	14.8	20.6	16.8	15.0	29.3
1-2 times/week	28.2	29.6	30.2	29.3	31.5	30.3	29.7	19.5	22.4	22.2	22.5	20.5
3-4 times/week	23.3	23.6	22.6	24.8	22.4	22.1	19.7	27.5	28.0	26.6	26.6	26.6
Everyday	23.2	18.9	16.5	20.5	17.3	13.6	12.9	38.3	29.0	34.4	35.9	23.6
Smoking status (%)												
Never	27.5	14.2	18.1	26.5	18.7	13.6	9.9	95.2	59.5	78.8	82.9	51.0
Past	15.0	29.9	20.1	21.3	21.8	20.4	15.2	0.9	11.7	3.6	3.2	6.1
1-19 cigarrets/day	13.2	16.4	15.7	16.3	16.8	15.4	13.3	2.8	18.8	12.3	10.6	24.1
20< cigarrets/day	43.3	39.6	46.1	35.9	42.8	50.6	61.6	1.1	10.0	5.2	3.2	18.8
Walking (%)												
1 hr/day≤	45.7	44.5	45.6	43.4	45.2	46.8	47.0	45.3	41.8	42.8	42.2	40.1
<1 hr/day	54.3	55.5	54.5	56.6	54.8	53.2	53.0	54.7	58.2	57.2	57.8	59.9

SD: standard deviation.

Table 2 presents RRs for all-cause mortality according to categories of alcohol consumption. For men, age-adjusted analysis showed that the risk of death was significantly higher in current drinkers than in never-drinkers, and that the risk increased linearly as the amount of alcohol intake increased. The risk was two times higher in past drinkers than in never-drinkers. These results remained basically unchanged after multivariate adjustment or after the exclusion of subjects who died during the first three years of follow-up. For women, age-adjusted analysis showed that the RR death was significantly higher in current heavy drinkers (consuming 22.8 g or more alcohol per day), but not in current moderate drinkers (consuming <22.8 g alcohol per day), as compared with never-drinkers. The risk was also higher in past drinkers than in never-drinkers. These results remained basically unchanged after multivariate adjustment or after the exclusion of subjects who died during the first three years of follow-up.

**Table 2.** Relative risks (RR) of all-cause mortality according to alcohol consumption in men and women.^†^

	Past drinkers	Never drinkers	Current drinkers (g alcohol per day)					P for trend
Men			All	<22.8	22.8-45.5	45.6-68.3	≥68.4	

Person-years	14,449	33,927	163,850	49,621	39,212	47,355	27,662	
No. of death	187	175	973	238	229	286	220	
Age-adjusted RR	2.16 (1.76 - 2.66)	1.00	1.29 (1.10 - 1.52)	1.07 (0.88 - 1.30)	1.22 (1.00 - 1.48)	1.26 (1.04 - 1.52)	1.92 (1.57 - 2.34)	<0.001
Multivariate RR1	1.86 (1.50 - 2.29)	1.00	1.21 (1.03 - 1.43)	1.10 (0.90 - 1.33)	1.17 (0.96 - 1.42)	1.16 (0.96 - 1.40)	1.62 (1.32 - 1.99)	<0.001
Multivariate RR2	1.86 (1.48 - 2.34)	1.00	1.21 (1.01 - 1.43)	1.09 (0.88 - 1.35)	1.14 (0.92 - 1.42)	1.20 (0.97 - 1.47)	1.59 (1.27 - 1.98)	<0.001

Women			All	<22.8	≥22.8			

Person-years	7,514	138,963	45,465	39,879	5,586			
No. of death	36	400	108	78	30			
Age-adjusted RR	1.78 (1.27 - 2.51)	1.00	1.09 (0.88 - 1.35)	0.90 (0.70 - 1.15)	2.43 (1.67 - 3.53)			<0.001
Multivariate RR1	1.40 (0.98 - 2.00)	1.00	1.01 (0.81 - 1.27)	0.87 (0.68 - 1.12)	1.98 (1.33 - 2.95)			0.005
Multivariate RR2	1.33 (0.88 - 2.00)	1.00	1.12 (0.88 - 1.43)	0.98 (0.75 - 1.28)	2.10 (1.36 - 3.24)			0.005

† : Adjusted for age in years; education (up to 15 years of age, 16-18, or 19 years or older); marital status at baseline (whether or not living with spouse); past histories of hypertension, renal diseases, liver diseases, diabetes mellitus, peptic ulcers, or tuberculosis; cigarette smoking (never smokers, past smokers, current smokers smoking 1-19 cigarettes per day, or current smokers smoking at least 20 cigarettes per day); body mass index in kg/m^2^ (less than 18.5, 18.5-24.9, or 25.0 or higher); walking time per day (less than 1 hour, or 1 hour or longer); consumption frequencies of green vegetables and oranges (almost daily, 3-4 times per week, 1-2 times per week, or 1-2 times per month or less often). Multivariate RR2 has been estimated with the exclusion of 317 subjects (220 men and 97 women) who died within the first 3 years of follow-up. Numbers in parentheses are 95% confidence intervals.

As shown in Table 3, the increased risk of all-cause mortality associated with current alcohol consumption was remarkable only among younger men (40-49 years of age) but not among older men (50-59 years of age and 60 years of age or older). We did not observe such differential findings by age groups for women. For other variables, we did not find substantial modification of the association between alcohol consumption and mortality (data not shown).

**Table 3.** Relative risks (RR) of all-cause mortality according to alcohol consumption by age classes in men.^†^

	Past drinkers	Never drinkers	Current drinkers (g alcohol per day)					P for trend

			All	<22.8	22.8-45.5	45.6-68.3	≥68.4	
40-49 years old								
Person-years	3,890	13,189	75,076	23,802	16,929	20,171	14,175	
No. of death	23	17	208	58	44	49	57	
Age-adjusted RR	4.62 (2.47 - 8.64)	1.00	2.16 (1.31 - 3.53)	1.90 (1.10 - 3.26)	2.02 (1.15 - 3.54)	1.89 (1.09 - 3.27)	3.14 (1.82 - 5.39)	<0.001
Multivariate RR1	3.60 (1.90 - 6.82)	1.00	2.10 (1.27 - 3.45)	1.97 (1.14 - 3.39)	2.01 (1.14 - 3.53)	1.83 (1.05 - 3.19)	2.74 (1.58 - 4.76)	0.001
Multivariate RR2	3.50 (1.72 - 7.12)	1.00	2.20 (1.27 - 3.81)	2.00 (1.10 - 3.64)	2.05 (1.11 - 3.82)	1.98 (1.08 - 3.65)	2.97 (1.63 - 5.42)	<0.001

50-59 years old								
Person-years	6,022	12,784	60,136	17,420	14,565	17,915	10,238	
No. of death	75	72	427	106	98	119	104	
Age-adjusted RR	2.23 (1.61 - 3.08)	1.00	1.26 (0.98 - 1.62)	1.08 (0.80 - 1.46)	1.20 (0.88 - 1.62)	1.18 (0.88 - 1.58)	1.81 (1.34 - 2.45)	<0.001
Multivariate RR1	1.82 (1.30 - 2.53)	1.00	1.20 (0.94 - 1.55)	1.14 (0.84 - 1.54)	1.16 (0.86 - 1.58)	1.12 (0.83 - 1.50)	1.50 (1.10 - 2.04)	0.019
Multivariate RR2	1.84 (1.28 - 2.65)	1.00	1.15 (0.87 - 1.52)	1.11 (0.80 - 1.54)	1.14 (0.81 - 1.59)	1.08 (0.78 - 1.50)	1.34 (0.95 - 1.90)	0.114

60 years old or older								
Person-years	4,537	7,954	28,637	8,399	7,718	9,270	3,250	
No. of death	89	86	338	74	87	118	59	
Age-adjusted RR	1.83 (1.36 - 2.47)	1.00	1.09 (0.86 - 1.38)	0.81 (0.60 - 1.11)	1.04 (0.77 - 1.40)	1.18 (0.89 - 1.56)	1.69 (1.21 - 2.35)	<0.001
Multivariate RR1	1.59 (1.17 - 2.15)	1.00	1.02 (0.80 - 1.30)	0.82 (0.60 - 1.12)	0.99 (0.73 - 1.34)	1.08 (0.81 - 1.43)	1.46 (1.04 - 2.05)	0.003
Multivariate RR2	1.61 (1.16 - 2.25)	1.00	1.06 (0.82 - 1.38)	0.86 (0.61 - 1.21)	0.95 (0.68 - 1.33)	1.17 (0.86 - 1.58)	1.51 (1.05 - 2.18)	0.002

† : Adjusted for education (up to 15 years of age, 16-18, or 19 years or older); marital status at baseline (whether or not living with spouse); past histories of hypertension, diabetes mellitus, renal diseases, liver diseases, peptic ulcers, or tuberculosis; cigarette smoking (never smokers, past smokers, current smokers smoking 1-19 cigarettes per day, or current smokers smoking at least 20 cigarettes per day); body mass index in kg/m^2^ (less than 18.5, 18.5-24.9, or 25.0 or higher); walking time per day (less than 1 hour, or 1 hour or longer); consumption frequencies of green vegetables and oranges (almost daily, 3-4 times per week, 1-2 times per week, or 1-2 times per month or less often). Multivariate RR2 has been estimated with the exclusion of 220 subjects who died within the first 3 years of follow-up. Numbers in parentheses are 95% confidence intervals.

## DISCUSSION

This prospective cohort study of the Japanese general population demonstrated a linear association between the amount of current alcohol consumption and the risk of all-cause mortality among men and women. We did not observe lower mortality among men and women who consumed moderate amount of alcohol (<22.8 g per day) as compared with never-drinkers.

The discrepancy between the present results and those of most previous studies showing a J-shaped^1^^-^^11^ or U-shaped^12^^-^^18^ association may be partly explained by the exclusion in this study of past drinkers from the reference category. In most previous studies, “non-drinkers” comprised both never drinkers and past drinkers. In the present analysis, we considered past drinkers and never drinkers separately, and found that past drinkers had markedly higher risk of all-cause mortality compared with never drinkers. Higher mortality among past drinkers may be due to ill health that had led them to quit drinking. Studies of alcohol and all-cause mortality may overestimate the lower risk in moderate drinkers if they did not separate never drinkers and past drinkers in the referent group.^22^

In this study, the increased risk of all-cause mortality associated with current alcohol consumption was more remarkable among younger men than among older men. These results are consistent with a large population study in England and Wales that found a steeper dose-response association between alcohol consumption and all-cause mortality among young men than among older men.^38^

Three studies have examined the association between alcohol consumption and all-cause mortality among Japanese men. Tsugane et al.^11^ followed up 19,231 men aged 40-59 years for 7 years and documented 548 deaths. Compared with nondrinkers which comprised both never drinkers and past drinkers, the authors observed significantly lower risk for men who consumed 1-149 g alcohol per week (RR, 0.64; 95% confidence interval [CI], 0.46-0.88), and significantly higher risk for men who consumed ≥ 450 g alcohol per week (RR, 1.32; 95% CI, 1.00-1.74). Kono et al.^27^ followed up 5,135 men aged 25 years or older for 19 years and documented 1,283 deaths. Compared with never drinkers separated from past drinkers, significantly higher risk was observed for men who consumed ≥2 go or 54 ml alcohol per day (RR, 1.28; 95% CI, 1.07-1.52). Miyazaki et al.^32^ followed up 6,652 men aged from 40 to 69 years for up to 11 years and ascertained 379 deaths. Compared with never drinkers separated from past drinkers, the authors observed significantly lower risk for men who were occasional drinkers (RR, 0.71; 95% CI, 0.50-0.99) and daily drinkers who consumed less than 25 g alcohol per day (RR, 0.51; 95% CI, 0.29-0.88), while they did not find significant increase in risk for men who consumed higher amount of alcohol.

The present study had some methodological advantages over previous studies of alcohol and mortality conducted in Japan.^11^^,^^27^^,^^32^ First, the present study had a larger number of deaths than did previous studies. Second, the validity and reproducibility of the questionnaire was not confirmed in some of the previous studies.^27^^,^^32^ In this study, we used questionnaire with high validity and reproducibility.^36^ Third, our study controlled extensively potentially confounding variables including age, smoking, education, body mass index, consumptions of orange or spinach, walking time, marital status, and past histories of chronic diseases.

To our knowledge, this is the first study examining the association between alcohol and all-cause mortality among Japanese women. Studies in Western populations have been inconsistent, reporting J-shaped,^6^^,^^10^^,^^18^^,^^33^ U-shaped,^34^ no,^28^^,^^31^ or other^16^ associations between alcohol consumption and all-cause mortality. We observed that, compared with never drinking, drinking 22.8 g of alcohol or more per day was associated with an increased risk for all-cause mortality, while drinking moderate amount of alcohol (<22.8 g per day) was not associated with a lower mortality.

In summary, based on 11 years follow-up of rural Japanese men and women, we found a higher risk of mortality in past drinkers and a linear association between the amount of current alcohol consumption and the risk of all-cause mortality among men as well as among women, and the association was remarkable for younger men.
